# Pharmacogenomics to Improve Supportive Care Symptoms. A Prospective Observational Study Protocol

**DOI:** 10.3310/nihropenres.14182.1

**Published:** 2025-12-22

**Authors:** Martyn Patel, John McDermott, William Newman, Caroline Barry

**Affiliations:** 1 Norfolk and Norwich University Hospital NHS Foundation Trust, Norwich, England, UK; 2 University of East Anglia Norwich Medical School, Norwich, England, UK; 3 Manchester University NHS Foundation Trust, Manchester Centre for Genomic Medicine, Manchester, England, UK; 4 Evolution, Infection and Genomics, School of Biological Sciences, The University of Manchester, Manchester, England, UK

**Keywords:** Supportive care; palliative care; medicines optimisation; pharmacogenomics; pharmacogenetics

## Abstract

**Background:**

People living with life limiting conditions such as incurable cancer often have problems with pain, vomiting and other symptoms which impact quality of life. Supportive and Palliative Care aims to improve people’s symptoms, particularly towards the very end of life. Many medications exist that can be used to help with these symptoms, but often they do not work, or they cause side effects for the people taking them. “Pharmacogenomics” is a way of predicting who is more likely to have a good response to drugs, and who is more likely to get side effects, based on variation in their genes.

**Methods:**

A prospective observational study, recruiting 50 patients aged over 18, with life limiting conditions. The study aims to understand how the introduction of multi-gene pharmacogenetic test for patients receiving palliative and supportive care might impact routine prescribing practice in an acute NHS hospital setting. It will report on the nature of prescribing patterns over a 90-day period post recruitment, looking for any variation between patients with and without the presence of actionable drug-gene interactions. It will help determine the future feasibility of incorporating pharmacogenomic testing in a palliative care population based on recruitment targets versus actual recruitment achieved. A drug-gene interaction ratio for this cohort will be reported, giving an indication of the scale of the use of medications relevant to pharmacogenomics used in palliative care, and the potential scope of future interventions. The study involves the collection of a single blood sample, and genetic results will not be shared with participants.

**Discussion:**

This single site study aims to provide preliminary data to inform study design of clinical trials that can definitively address the question of clinical utility in this population, whilst also understanding the acceptability of researching this topic in this specific patient population.

**Trial registration:**

**ClinicalTrials.gov ID** NCT06856122, registered 2025-03-04

URL: https://clinicaltrials.gov/study/NCT06856122

## Plain Language Summary

People living with terminal or life limiting conditions such as incurable cancer often have problems with pain, vomiting and other symptoms which impact quality of life. Supportive and Palliative Care aims to improve the symptoms of people with terminal conditions, particularly towards the very end of life. There are many drugs that can be used to help with these symptoms, but often they do not work, or they cause side effects for the people taking them. “Pharmacogenomics” is a way of predicting who is more likely to have a good response to drugs, and who is more likely to get side effects, based on their genes. We know that changes in certain genes (as happens naturally in the population) might mean that a drug might not work as well for some people as others. We are interested in whether testing for these differences in genes for people living with cancer and experiencing symptoms, might help improve their care, and how to provide feedback from these test results to the clinicians involved in patient care.

## Background

Many people living with serious, incurable illnesses will experience problems with pain, nausea, vomiting and other symptoms that affect their quality of life. Where these symptoms are associated with the treatment of life limiting conditions, it may affect how likely someone is to take those medications, or lead to further complications. Palliative and supportive care aims to relieve or reduce symptoms arising from serious illness or its treatment.

Commonly, this involves the use of opioids and non-steroidal anti-inflammatory drugs for pain management, anti-emetics for nausea and vomiting and a myriad of other supportive care medications (SCM). Symptom control in this patient cohort can be challenging due to varying responses to medication, polypharmacy burden/known drug-drug interactions, and frailty or co-existing comorbidities. Up to a third of bereaved relatives report inadequate symptom control after a hospital death ^1^. Complaints about the quality of pain management towards the end of life are common ^2^. With an estimated 20 people a day dying in pain in hospitals across the UK ^3^.

Pharmacogenomics (PGx) is an area of expanding research, which could indicate whether an individual is likely to benefit from a SCM, based on their genetic profile. PGx can identify genetic variations that influence the metabolism of certain medications, leading to variations in effects between individuals. Most people carry at least one pharmacogenetic variant that could influence their response to a medicine ^4^.

Common variation in an individual’s genetic code can lead to functional variation in the expressed proteins, including enzymes and drug transporters ^5^. This is particularly relevant in the cytochrome P450 family of genes, where variation can lead to the production of enzymes with differential activity profiles. For example, genetic variation in the *CYP219* gene can impact the activity of the cytochrome P450-2C19 (CYP2C19) protein. This leads to a spectrum of potential phenotypes, ranging from poor metabolisers (PM), intermediate metabolisers (IM), normal metabolisers (NM), rapid metabolisers (RM) to ultra-rapid metabolisers (UM) of various medications.

It is now possible to perform genetic testing to predict an individual’s functional phenotype for a given pharmacogene. This information can then be used to guide prescribing for a wide range of medications against published expert advice ^6^. Single-gene tests are currently available in many health systems around the world to guide prescribing, including the NHS where all patients receiving fluoropyrimidine chemotherapy agents or mavacamten must have pharmacogenetic testing prior to prescription ^7^. In recent years, many health systems have begun to explore the use of panel based genetic testing, which analyses variants across multiple genes at the same time. However, these are comparatively rarely used in the United Kingdom (UK) despite the potential economic impact ^8^.

Many of the medications used in palliative and supportive care may be susceptible to drug-gene interactions, which may determine how effective those medications are at achieving symptom control and a “good death”. This is particularly important in a palliative care context, where achieving adequate pain or symptom management in a timely manner is often the overriding therapeutic aim.

The existing evidence base for PGx predominantly concerns the medicines prescribed in cardiology, stroke, psychiatry and oncology ^6^. This evidence originates from trials conducted mostly outside of the UK, with different funding models and therefore health economic impact calculations that are less relevant to National Health Service (NHS) practice ^9, 10^. Our recent review into pharmacogenomics and symptom management in palliative care found that pharmacogenomic testing, to identify drug-gene interactions was feasible in palliative care setting (predominantly North America) ^11^. Many drugs used commonly in palliative care were amenable to a pharmacogenomic approach, with data suggesting that PGx information might result in a change in prescribing advice in around a quarter of cases ^12^.

It would seem a logical extension from this point to suggest that PGx therefore could improve symptom control in supportive and palliative care, but to date no trials have been reported in the UK or Europe that could answer either the potential drug-gene interaction ratio in this patient population, nor the feasibility of doing PGx testing in a UK supportive/palliative care setting. Without this data, it is impossible to calculate the clinical utility of adopting such an approach. ‘Clinical utility’ often refers to the likelihood that a test will, by prompting an intervention, result in an improved health outcome. In this context, clinical utility might specifically refer to improved symptom management from prescribing changes informed by an identified drug-gene interaction. Addressing the paucity of evidence around real-world clinical utility is an essential step in policy and practice development ^13^.

The Pharmacogenomics to Improve Supportive Care Symptoms (PISCES) study aims to understand the acceptability and feasibility of recruiting NHS patients into a pharmacogenomics research study in a palliative and supportive care context, and also to provide preliminary data on drug-gene interaction prevalence (how often a potentially actionable genetic variation is found paired to an existing prescription of a medication in the same patient). We aim to use data to inform study design of future clinical trials that can definitively address the question of clinical utility in this population.

## Methods/design

This is a prospective, observational cross-sectional study of adult patients with serious and/or life limiting conditions, such as incurable cancer undergoing palliative or supportive care treatment.

**Ethical consent statement:** All participants in this study will undergo written informed consent.

Participants will receive the usual standard of care for their condition, and from enrolment will have all their medication use recorded for a 90-day period. They will have a single blood test that will be analysed for *CYP2D6*, *CYP2C19* and *CYP2C9* variants. This genetic analysis will take place after study data collection for medication use is complete. We will use this data to ascertain whether there were any clinically actionable drug gene interactions for a subset of medications used in supportive and palliative care treatment compared with their medication usage at enrolment (see Pharmacogenomic analysis section below for explanation of difference between “clinically relevant” and “clinically actionable” results).

We will adhere to the STrengthening the Reporting of OBservational studies in Epidemiology (STROBE) guidelines for reporting observational cohort studies - https://www.strobe-statement.org/

### Patient and Public Involvement

We have conducted Patient and Public Involvement (PPI) workshops to seek feedback on conducting research in this area in this patient group. All PPI attendees had personal experience of palliative care delivered to someone in their family. They gave universal support for the idea of doing research generally in palliative care setting and they specifically felt there was value for learning more about the utility of pharmacogenomic testing in general.

## Aims and objectives

**Primary aim** (or Research Question)

*How could the introduction of multi-gene pharmacogenetic test for patients receiving palliative and supportive care could impact routine prescribing practice in an acute NHS hospital setting?*

Primary and secondary outcome measures are illustrated in Table 1, and the objectives behind these described below in more detail.

**Table 1.** Outcome measures.

Outcome Measure	Measure Description	Time Frame
Drug-gene interaction ratio (DGI) (primary outcome)	The total number of clinically actionable drug-gene pairs (genetic variation results that pair with a currently prescribed medication for that same individual), divided by total number of individuals in cohort.	This will be calculated for all medications prescribed at the point of recruitment.
Frequency of changes in prescription medication that are potentially affected by drug-gene interaction (secondary outcome)	Number of participants who have a change in prescribed medication (dose or formulation) during 90-day follow up period that could be theoretically paired to possession of a clinically actionable drug-gene interaction	From enrolment until 90 days post blood test

**Primary Objective:**

- To understand the clinical utility of multi-gene pharmacogenetic testing in patients receiving palliative and supportive care across palliative care settings (inpatient hospital, outpatient), specifically by calculating a drug-gene interaction (DGI) ratio, based on extant prescriptions at time of recruitment paired with an individual’s “clinically actionable” pharmacogenetic results. We will also report the proportion of subjects that have at least one clinically actionable DGI out of all subjects in the study.

**Secondary Objective:**

- To report on the nature and variation of prescribing patterns between patients with and without the presence of clinically actionable drug-gene interactions
- To report on the feasibility of incorporating PGx testing in a supportive and palliative care setting (in an acute hospital setting) via achievement of recruitment targets

### Inclusion criteria

- Aged 18 years or older
- Confirmed life-threatening or life limiting illness (examples including but not limited to stage 4 cancer diagnosis, motor neurone disease, end stage renal failure) as judged by named clinician in charge of care or consulting palliative care clinician.

### Exclusion criteria

- Lacks capacity to decide on research participation/ is unable to consent to research participation due to impaired mental capacity
- Prognosis likely less than one week (as judged by named clinician in charge of care)
- Enrolment in clinical trial of a supportive care / symptom management medication (those on clinical trials for disease modifying cancer therapy can be included).
- Under 18 years old

### Study process

Patient recruitment is expected to take place between May 2025 and November 2025 at the Norfolk and Norwich University Hospitals NHS Trust (NNUH). Individuals referred to the in-patient palliative care service at NNUH or attending out-patients with oncology or palliative care will be informed about the study by their clinician, or a supporting health-care professional, and offered a short form patient information sheet. Patients who are interested in joining the study will be asked for permission to share their contact details with the study team. This will be done verbally, with site staff sending an electronic or paper notification of consent to contact to the research team. On receipt of referral, a trained member of research team member will visit in person (if still an in-patient) or telephone potential participants to explain the study. Following this, the team will send a long form patient information sheet to potential participants.

If the participant would like to join the study, the research team will arrange a study visit (either as an in-patient, or to coincide with next out-patient attendance) for formal consenting and obtaining a 5ml blood sample. We will record the numbers of potential participants who decline participation, and any reasons given, where possible. No financial or non-financial incentives are offered to participants.

All participants will undergo testing of a panel of genetic variants linked with medications commonly used in symptom control for which there is internationally recognised guidance on PGx testing (see Table 2) ^14^. This will involve collecting a 5mL blood sample from individuals. All participants will be consented to examination of their records within local hospitals and/or primary care to extract study relevant data on prescribing.

**Table 2.** Drug/Gene panel.

Gene	Relevant Drug
*CYP2D6*	Ondansetron, oxycodone, tramadol, hydrocodone, codeine, methadone, cyclizine, tropisetron, nortriptyline, venlafaxine, metoclopramide
*CYP2C19*	Amitriptyline, Citalopram, escitalopram, omeprazole, pantoprazole, diazepam
*CYP2C9*	Celecoxib, meloxicam, piroxicam, ibuprofen

The start of the 90-day follow-up will be from the date of the blood sample. Standard demographic information including ethnicity will be collected at baseline.

Pharmacogenomics assay

DNA will be extracted from the participant blood samples in an ISO15189 accredited laboratory (NHSE NW Genomics Lab Hub) at Manchester Centre for Genomic Medicine, Manchester University NHS Foundation Trust. We will use the Agena VeriDose® Core Panel v2.0 panel, plus digital PCR assay for *CYP2D6* genotyping. Both assays have been used in the NHS PROGRESS study (ISRCTN15390784). We may set up an alternative assay(s) (dependent upon analytical validity, cost and ease of use if this becomes available), which covers the same genetic variants. No other genetic tests will be performed without consent. Unused DNA samples will be returned to NNUH on completion of the study.

### Pharmacogenomic analysis

We will analyse the results of a number of variants in CYP2C9, CYP2C19 and CYP2D6, as variants in these three genes are connected to the predicted response to drugs used in palliative symptom control and have specific internationally agreed CPIC guidance ^14^. Therefore, defined actions can be recommended dependent upon the results namely: CYP2C19 - proton pump inhibitors, CYP2C9 – NSAIDs; and CYP2D6 – ondansetron, tropisetron and opioids.

The potential impact of pharmacogenetic testing, had the data been available at the point of prescribing, will be categorized as either ‘no impact’, ‘clinically relevant’ or ‘clinically actionable’ based on CPIC guidance and previously published definitions ^15^. A ‘clinically actionable’ interaction is defined as one where the recommended action within current CPIC guidance is to either alter the chosen medicine or alter the dose of the same medicine. A ‘clinically relevant’ interaction is where the gene–drug interaction conferred increased risk of an adverse drug reaction (ADR) or reduced effectiveness, though the recommended action was to initiate therapy at a standard dose. Our primary outcome will be calculated based on clinically actionable results only.

### Data analysis plan

Baseline characteristics of each participant will be aggregated and summarised, including age, sex, allergy status, ethnicity, diagnosis, and medications prescribed. For categorical variables, the number and percentage will be presented. For continuous variables, the mean (and standard deviation) or median (and interquartile range) will be presented depending on the distribution.

We will report data on our primary and secondary outcome measures as detailed above, and we will also report data on:

1. Number of participants with at least one clinically actionable genetic variant related to a medication they were prescribed, and proportions of participants with at least one clinically actionable or clinically relevant result vs those with zero.
2. Estimated number of patients where the genetic variant result could influence future prescribing

Further details on feasibility and acceptability of any future interventional trial will be evaluated by:

- Number of potentially eligible participants who meet the inclusion criteria
- Number of participants subsequently recruited into the study
- % of patients where full prescribing data can be accessed by digital only methods to understanding prescribing patterns over acute, primary and hospice care.
- % of patients with incomplete pharmacogenetic data (where some genetic variants have failed) due to low DNA yield from blood sampling or other technical factors.

## Discussion

### Ethical considerations

In designing the study, we have considered the potential disadvantages for participation for people living with incurable illness, and whether a further observational study into PGx is justified given the existing international evidence base. At present, within the UK there is clinical equipoise as to whether PGx testing is likely to change clinical outcomes. Furthermore, there is limited European data as to the acceptability of testing in this population. This study gives up the opportunity to learn about the acceptability of PGx testing for those approaching the end of life, and we will report on screening/recruitment rates to understand how acceptable our approach is.

As this is an observational study, we will make it clear that there is no plan to feedback results to individual participants, and that all participants will receive standard care. We will however offer the opportunity to receive a lay summary of the overall results and impact of the study.

### Ethical approval

This was granted by London-Surrey Research Ethics Committee, 10 ^th^ March 2025, IRAS project ID: 354053. All participants in this study will undergo written informed consent.

### Limitations

Limitations of the study are common to many single site, observational studies of this nature. Findings arising from this study may not be generalisable across all palliative care populations. The study site is an area of low ethnic diversity and there we anticipate that our study sample may not be fully representative of the population. We will try and purposively sample people from non-white backgrounds were possible to redress this.

A further limitation is absence of retrospective data collection on medicines use. Whilst the results will capture both current and future SCM, it will not consider medications that have previously been tried and subsequently stopped either due to ineffectiveness or side effects. This may mean that our results are not fully reflective of the potential clinical utility of PGx testing throughout the illness journey.

### Addendum

A preprint version of this protocol is available at : ^16^

https://www.researchsquare.com/article/rs-7723169/v1

### Consent to participate declaration

Ethical approval has been granted by London-Surrey Research Ethics Committee, 10 ^th^ March 2025, IRAS project ID: 354053.

### Consent to publish declaration

Not applicable.

### List of abbreviations

CQUIN - Commissioning for Quality and Innovation

PGx - pharmacogenomics

SCM - supportive care medications

PM – poor metaboliser

IM – intermediate metaboliser

NM – normal metaboliser

UM – ultra metaboliser

NHS - National Health Service

UK – United Kingdom

PISCES - Pharmacogenomics to Improve Supportive Care Symptoms

PPI – Patient and Public Involvement

NNUH - Norfolk and Norwich University Hospitals NHS Trust

## Data Availability

Data sharing is not applicable to this article as no datasets were generated or analysed during the writing of this protocol, i.e. “No data are associated with this article.
