# Supplementary material for: Pharmacogenomics to Improve Supportive Care Symptoms. A Prospective Observational Study Protocol
Source: NIHR Open Res. 2026 Apr 10;5:125. Originally published 2025 Dec 22. [Version 2] doi: 10.3310/nihropenres.14182.2 (PMC13096778; doi:10.3310/nihropenres.14182.2)
Supplement: Peer review history

# Reviewer report: Reviewer response for version 2

**DOI:** 10.3310/nihropenres.15571.r40326

**Published:** 2026-04-20

**Authors:** Andrew Somogyi

**Affiliations:** 1 Faculty of Health and Medical Sciences, Adelaide, Australia; 2 Pharmacology, Adelaide University (Ringgold ID: 103037), Adelaide, South Australia, Australia

Significant improvements have been made and all my suggestions have been adequately addressed.

Is the study design appropriate for the research question?

Partly

Is the rationale for, and objectives of, the study clearly described?

Yes

Are sufficient details of the methods provided to allow replication by others?

Partly

Are the datasets clearly presented in a useable and accessible format?

Yes

Reviewer Expertise:

Pharmacogenomics in palliative medicine

I confirm that I have read this submission and believe that I have an appropriate level of expertise to confirm that it is of an acceptable scientific standard.

# Reviewer report: Reviewer response for version 2

**DOI:** 10.3310/nihropenres.15571.r40327

**Published:** 2026-04-11

**Authors:** Victoria Pratt

**Affiliations:** 1 Indiana University School of Medicine, Indianapolis, Indiana, USA

My concerns have been adequately addressed.

Is the study design appropriate for the research question?

Partly

Is the rationale for, and objectives of, the study clearly described?

Yes

Are sufficient details of the methods provided to allow replication by others?

Yes

Are the datasets clearly presented in a useable and accessible format?

Yes

Reviewer Expertise:

Pan-ethnic pharmacogenetic testing.  PGx implementation.

I confirm that I have read this submission and believe that I have an appropriate level of expertise to confirm that it is of an acceptable scientific standard.

# Reviewer report: Reviewer response for version 1

**DOI:** 10.3310/nihropenres.15438.r39369

**Published:** 2026-02-12

**Authors:** Andrew Somogyi

**Affiliations:** 1 Faculty of Health and Medical Sciences, Adelaide, Australia; 2 Pharmacology, Adelaide University (Ringgold ID: 103037), Adelaide, South Australia, Australia

A reasonably well-written protocol designed to pilot a potential future study in a cohort of patients in palliative & supporting care, for whom pharmacogenomic testing and outcomes (acceptability, feasibility and implementation) has not been evaluated.

 Apart from the drug-drug interactions concern that can lead to false phenotyping and should be considered, there are a few other issues that could be addressed.

 That pain and nausea/vomiting are the only symptoms to be addressed from a medications perspective, but fatigue (antidepressants/diazepam) and constipation (often from opioids) could be considered as they are common.

 Prescribing patterns are from enrolment to 90 days. In such patient cohorts, SCM doses can be changed and drugs switched within the 90 days and those data and reasons for such changes appear not being documented.

 CPIC guidelines will be used for evaluation to address the aims as per Table 2; however, the evidence for CYP2D6 phenotype based on genotype altering oxycodone dosage per se shows lack of evidence and CYP2D6 only relates to codeine and tramadol  which are infrequently used in palliative care. Also, methadone is predominantly metabolised by CYP2B6 (especially the S-enantiomer) and CPIC guidelines  do not support a change in dosage.

 Given that medication side-effects are an important criterion for pharmacogenomic testing and that the planned patient population will be on multiple medications that may have a pharmacogenomic footprint, medication side effects should be considered.

Is the study design appropriate for the research question?

Partly

Is the rationale for, and objectives of, the study clearly described?

Yes

Are sufficient details of the methods provided to allow replication by others?

Partly

Are the datasets clearly presented in a useable and accessible format?

Yes

Reviewer Expertise:

Pharmacogenomics in palliative medicine

I confirm that I have read this submission and believe that I have an appropriate level of expertise to confirm that it is of an acceptable scientific standard, however I have significant reservations, as outlined above.

## References

[1] : Clinical Pharmacogenetics Implementation Consortium Guideline forCYP2D6 ,OPRM1 , andCOMT Genotypes and Select Opioid Therapy. *Clinical Pharmacology & Therapeutics* .2021;110(4) : 10.1002/cpt.2149 888-896 10.1002/cpt.2149 PMC824947833387367

[2] : Clinical Pharmacogenetics Implementation Consortium Guideline forCYP2B6 Genotype and Methadone Therapy. *Clinical Pharmacology & Therapeutics* .2024;116(4) : 10.1002/cpt.3338 932-938 10.1002/cpt.3338 PMC1158719338863207

# Response

**Published:** 2026-03-19

**Authors:** Martyn Patel

**Affiliations:** Norfolk and Norwich University Hospital, Norwich, England, UK

REVIEWER 2

- Thank you for acknowledging the importance of this topic
- We have expanded on the nature of side effects from SCM in the introduction
- With regards to patterns in medication changes over the 90 day period, we are committed to checking for the reasons for switching of medications, and will document this where that information is available. One of our main feasibility questions is whether we can gather sufficient information about medicines changes from e-records alone, to avoid having to ask patients to fill in medication diary cards, making a future trial less burdensome. If this is not possible, that will itself be useful information. We have now amended the Limitations section to acknowledge the potential weakness of our current approach to side effect data collection in the limitations section.
- We are aware that the evidence base for pharmacogenomic prescribing guidance and opiates has weakened over time. This is why we chose a “panel” test approach to cover medication across different classes. We feel it will still be of interest to collect information on strong opiate related genetic variation, even if it currently does not impact prescribing as per CPIC guidelines. Our patient and public involvement groups were keen on multiple symptoms being considered within this study – we will make this clearer in the revised text.
- In our UK based experience patients are still sometimes prescribed “weak opiates” such as codeine and tramadol, but we acknowledge there is variance in this internationally. We will report on the nature of opiate prescribing in this cohort, which again will influence future trial design.
- We have removed methadone from our table of medications, thank you for pointing this out, and will consider CYP2B6 testing in future trials.
- With regards to capturing medication side effects. As mentioned, we are aiming for a light touch patient data collection, to encourage/enable participation. We have found that when prescriptions are changed, the specialist team letter to the primary care team does often mention side effects that led to the change, and we are noting these. Again, we will report if this is insufficient for our needs.

# Reviewer report: Reviewer response for version 1

**DOI:** 10.3310/nihropenres.15438.r38978

**Published:** 2026-01-07

**Authors:** Victoria Pratt

**Affiliations:** 1 Indiana University School of Medicine, Indianapolis, Indiana, USA

Overall, this is a well-written manuscript.  I have one major concern related to the study design.  The authors plan to evaluate drug-gene interactions, but do not plan to study drug-drug-gene interactions.  Patients receiving palliative or support care are often on many medications which can impact the predicted phenotype based on the genotype alone.  

 There are a few minor points.

 The authors use the word "carry".  While derived from genetics where a carrier of a pathogenic variant typically for an autosomal recessive disorder does not clinical symptoms.  Though this term, carrier, is pervasive in pharmacogenetics, it would be better to use the term have/having as individuals with a pharmacogenetic variant often will have a phenotype.  

 PGx testing helps predict an individual's phenotype.  Drug-drug-gene interactions can alter a predicted phenotype.  

 Please check spelling of neurone

 Please check if 1st person is permittable

 Please use HGVS nomenclature.  All genes should be italicized.  

 The study refers to a past date.  Please confirm and update tense, if applicable.

Is the study design appropriate for the research question?

Partly

Is the rationale for, and objectives of, the study clearly described?

Yes

Are sufficient details of the methods provided to allow replication by others?

Yes

Are the datasets clearly presented in a useable and accessible format?

Yes

Reviewer Expertise:

Pan-ethnic pharmacogenetic testing.  PGx implementation.

I confirm that I have read this submission and believe that I have an appropriate level of expertise to confirm that it is of an acceptable scientific standard, however I have significant reservations, as outlined above.

# Response

**Published:** 2026-03-19

**Authors:** Martyn Patel

**Affiliations:** Norfolk and Norwich University Hospital, Norwich, England, UK

**Response to peer review reports**

 REVIEWER 1

- We have added to our analysis plan to state we will also seek to identify the prevalence of potential drug-drug-gene interactions in this cohort.
- We have changed “carry” to “have” as suggested.
- We have added a sentence to reflect that drug-drug-gene interactions can potentially impact on phenotype.
- It can be either neuron or neurone, with the latter most used in the UK, see this article –

https://academic.oup.com/brain/article/143/1/374/5679563

- Have italicised all genes in the text.
- The dates for study commencement are correct, and it is conventionally permissible to publish the protocol after commencement, as long as it is before study activities are complete. This is due to the inherent time lag from first attempt to publish this protocol, to success.
